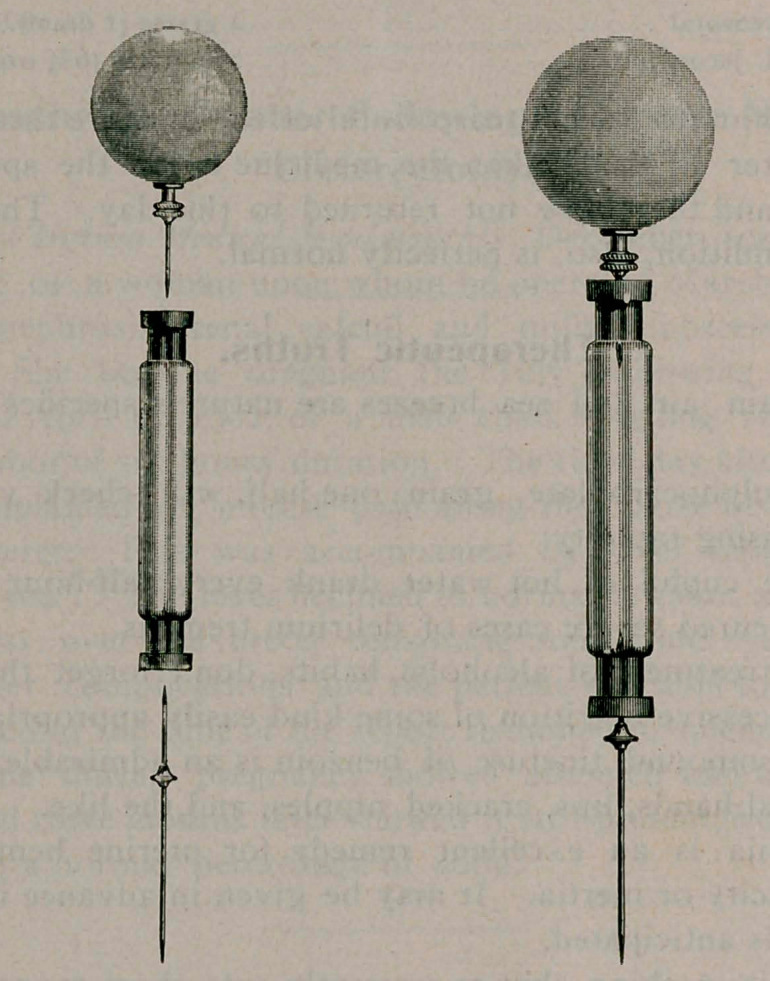# The Lynch Aseptic Syringe Container

**Published:** 1903-03

**Authors:** 


### NEW INSTRUMENTS.

### The Lynch Aseptic Syringe Container.

The above are illustrations of the Lynch aseptic syringe container, an original device for storing and ministering serum, and more particularly diphtheria antitoxin. The barrel of the syringe is filled with serum in the laboratory and aseptically sealed with rubber stoppers. The serum is never again exposed, nor is breakage, with its attendant danger of particles of glass, likely to happen. The needles and rubber bulb (one of each accompanying every dose of serum) are supplied in sterile wrappings, as will be seen by the illustrations; it is only necessary for the physician to pierce the stoppers with the needles to have a perfect hypodermic syringe. A feature of the device is that half of the contents of the container may be administered, when, by withdrawing the needles the apertures in the stoppers will close, keeping the remaining serum intact and sterile.

Dr. H. M. Alexander & Co. have adopted this device as a carrier for their diphtheria antitoxin, which is also supplied by Messrs. John Wyeth & Bro., as authorised general distributors.

## Figures and Tables

**Figure f1:**